# Domestic

**Published:** 1841-12-25

**Authors:** 


### DOMESTIC.

We quote from the N. Y. Medical Gazette for December, the following case described as fungus hæmatodes, chiefly because it furnishes a clear account of the condition of the lungs at a very advanced period in the progress of those changes induced in the thorax, towards the fatal conclusion of the peculiar disease described. Death generally follows amputation in this affection, before the morbid alterations of the thorax have reached such high perfection. The case will have additional interest for our older subscribers, in consequence of its parallelism with one evidently identical in the character of the disease reported by our friend, J. M. Wallace, M. D., then House Surgeon of the Pennsylvania Hospital. An account of the amputation—performed by Jacob Randolph, M. D., at that institution—together with its immediate consequences, will be found in our first volume, page 101, and the autopsy, performed about ten months after the operation, in the private practice of Dr. Wallace, is described at page 326 of the same volume.

It seems to us that, in Dr. Wallace's case, the progress of the tumor in the chest appears to have been arrested by death at an earlier period than in that reported by Dr. Markoe—

the encephaloid matter having become chiefly solved or broken down in the latter case, while in the former it constituted the mass of the diseased structure. The description by Dr. M. of the condition of the limb prior to amputation, is somewhat more full and satisfactory than that given by Dr. W.; the latter not having noted the condition of the veins on the superfices of the tumor, *if, indeed, they were really enlarged.* This varicosity, together with the regular encysted character of the tumors—both primary and consecutive—seem diagnostic in this form of disease, while the existence of bony spicula in both cases, and the altered texture of the femur, prove its accidental periosteal origin.

We have seen several cases of precisely this character, all terminating mortally, without operation, but have never been convinced that the disease is identical with that form of tumor equally ranked under the head of fungus hæmatodes, which sometimes seems to have a purely vascular origin, or takes its rise in an ecchymosis, and *walks regularly through all barriers of tissue*, regardless of their nature. The diagnosis of fungus hæmatodes is so obscure that, until informed of the peculiar views of the writer, we hardly know what idea to attach to the word when we meet with it in surgical works. The term "soft cancer," so often applied to one set of cases, could not be applied with any propriety to those alluded to in this article, which form a distinctly marked natural group, though whether they are *essentially* different from many others (those of the antrum, for instance) usually included under the head of fungus hæmatodes, can only be determined by the observations of the contemporary changes of structure in different organs of the same individual. A mass of such observations on the stomach, liver, lungs, &c., when affected with malignant alterations of structure, appearing simultaneously with, or as an obvious sequence of well known external tumors, would enable us to clear our diagnosis of constitutional surgical diseases, by distinguishing between the mere modifying influences of the structure of tissues and essential differences of vital action. In this point of view, both the cases to which reference has been made are deeply interesting.

R. C.

*Fungus Hæmatodes of the Thigh—Amputation—Return of the disease in the Chest.* By T. M. Markoe, M. D., House Surgeon N. Y. Hospital.—Joseph Peabody, aged 20, a native of Rhode Island, farmer, was admitted in the N. Y. Hospital, Oct. 6, 1841, with a large tumour on the left thigh, just above the knee-joint: of its origin he gave the following account. About eight weeks before his admission he laid down, while much heated by exercise, on some damp hay, and fell asleep. When he awoke he felt a sensation of stiffness about his right knee-joint, followed, in a short time, by a swelling on the upper and inner part of the knee, attended with some pain. This did not attract his particular attention for two or three weeks, during which time he made a journey from Wisconsin, where he was first attacked, to New York. Soon after his arrival here the swelling increased very rapidly, and was attended with a great deal of pain, so much that he was completely disabled. He then, by the advice of a physician, had the joint leeched several times, and put himself under the care of several different quacks in this city, without any benefit. The disease steadily advanced, his sufferings becoming so great as completely to deprive him of sleep. His health and strength failed rapidly, and his constitution seemed to be giving way under the violence of the disease. When admitted to the hospital he was pale and emaciated, with an anxious expression of countenance, strongly indicative of some severe disease. The tumour was situated on the outer and posterior parts of the right thigh, immediately above the knee-joint. At its largest part the diseased thigh measured 21 inches, while the sound one measured only 13. The tumour was quite firm to the feel, though with an obscure sense of fluctuation in some parts, apparently lobulated in its form and firmly fixed to the bone. Some parts of its surface were of a livid hue, with large veins traversing it in all directions. It was excessively tender to the touch, and he suffered in it, even when not handled, paroxysms of most excruciating pain. These pains were of a lancinating character, and confined to the tumour itself. The surface of the tumour was tense and shining, and its temperature was considerably above the natural standard. The foot and leg were œdematous, and the glands in the groin somewhat swollen and tender.

At a consultation of the surgeons, the disease was pronounced at once to be of a malignant character, and amputation was recommended as presenting the only chance of saving, or even of prolonging his life. The limb was accordingly removed at about the middle of the thigh, by Dr. Cheesman, on the 7th of October.

On laying open the tumour, it was found to consist, in part, of irregular cavities, containing a dirty brownish red fluid, and in other parts presenting an appearance like the substance of the brain, containing innumerable clots of blood of different sizes. It was firmly attached to the bone, which was itself softened and partly disorganized, and of a dark purple colour in the neighbourhood of the tumour. The whole was contained in a sac forming various lobes, generally not adherent to the surrounding tissues. Some parts of the muscular fibre which were most closely attached to the tumour, were beginning to be changed into a white fatty material, with obliteration of the original fibrous texture. Spiculæ of bone were felt here and there throughout the mass, more particularly in the fibrous envelope.

No unfavourable symptoms followed the amputation. The stump healed rapidly, and the patient regained his strength and spirits. His appetite returned, and he began to gain flesh. By the first of November he had recovered so far as to be able to leave his bed and sit up during the greater part of the day.

About this time, however, unfavourable symptoms began to appear. He complained of flying pains in his chest, accompanied by some dyspnœa and a short cough. The pulse became habitually frequent, and very often at evening his skin would be hot and dry. At the same time he became much dispirited and anxious about his prospect of recovery. His condition was not at all improved by the course of treatment adopted, which was moderately tonic, with occasional laxatives and counter-irritation to the chest. On the contrary, he failed rapidly. The cough almost entirely ceased, but his respiration soon became excessively embarrassed; indeed his principal complaint was of a sense of stricture in the lower part of the chest—which rendered a full expansion of the chest impossible. This was accompanied by palpitation of the heart, on the least exertion. The physical signs, on examination of the chest, were complete dulness on percussion, over the whole left side, and entire absence of respiration. The heart appeared to be displaced downwards and towards the median line, so that its pulsations were most distinctly felt in the epigastrium. The upper part of the abdomen, just below the edges of the ribs, was tense and hard, and perfectly dull on percussion. The lower part was without distension or dulness. With all this disease going on in the chest, the pulse was not at any time much excited, but was becoming more and more feeble every day, and his strength was rapidly failing. What tended most to reduce him was the profuse night sweat from which he suffered every night, and after which he always appeared much exhausted. Anodynes were the only remedies which gave him any relief from his sufferings, and he was accordingly kept constantly under their influence. He died apparently suffocated, Dec. 3d, 1840, a little less than two months after the amputation, and about four months from the commencement of the disease.

*Post Mortem* 18 hours after death.—On laying open the abdomen, the diaphragm on the left side was found pushed down, and forming a pouch reaching nearly to the umbilicus. This proved to be one of the walls of an immense cavity, bounded above by the lung and sides of the chest, and containing full a gallon of reddish brown watery fluid. The wall of this cavity presented a mellanated appearance. Rounded, flattened bodies, of a size from a pea to a hickorynut, and generally of a yellowish colour, lined the greater part of the cavity. These bodies were found to consist of a homogeneous soft brain-like matter, breaking down on very slight pressure. In various parts of the cavity, but principally at its lower portion, there existed numerous thin layers of soft semi-organized membrane of a dark red colour. In other parts the deposite was of a darker shade than that described above, and of a softer texture, and containing numerous points of coagulated blood. The lung of this side was pushed up to the upper part of the chest, and was every where closely adherent to the *pleura costalis.* Indeed no part of the original cavity of the pleura existed. The whole substance of the lung was converted into a soft, friable, reddish-white mass, in which no traces of the original texture could be observed except a slight striated appearance of the surface, and its breaking into pretty regular flakes on attempting to raise it. The pleura and apparently the sub-pleural cellular tissue, in many parts, particularly along the spine, were converted into a tissue of a chalky feel, friable, and of a whitish colour. This deposite was more particularly abundant in the upper part of the chest, and was the means of an almost inseparable union between the lung and the walls of the chest. On the surface of the other lung were several spots, from the size of a five-pence to that of a shilling, piece where the pleuræ were adherent, in some of which the same chalky matter above described existed. Near the root of this lung was a mass half as large as an egg, of the same soft texture as that into which the left lung was entirely converted. The heart was healthy, but much displaced downwards and inwards.—The abdominal organs presented no trace of the disease. The stump was particularly examined, and the glands in the groin above, both were apparently perfectly sound.—*N. Y. Medical Gazette.*

---

We have much pleasure in complying with a request of our friend, Professor Dunglison, to publish the subjoined correspondence in defence of a distinguished English physician from an undeserved imputation which has been cast upon him in this country. It appears that

Dr. Paine, of New York, author of Medical and Physiological Commentaries, conceived that an unfavourable review of his work in the British and Foreign Medical Review had proceeded from the pen of Dr. Carpenter, of Bristol. In a reply to this *critique*, Dr. Paine felt himself warranted, not only in treating Dr. Carpenter as the author of it, but in fathering upon that gentleman a previous article in the same review, which contained a plagiarism from the writings of Dr. Channing. This imputation upon Dr. Carpenter has, we are informed, been extensively circulated in this country, and it is but justice to give currency to his refutation. We have no doubt that Dr. Paine will, if opportunity offer, be happy to unite in the correction of an error into which he has been thus hastily led.

*Copy of a Letter from Dr.* W. B. Carpenter, *of Bristol (England), to Professor* Dunglison, *of Philadelphia, in reference to certain charges made against the former, by Dr.* Martin Paine, *Professor of the Institutes of Medicine in the University of New York, in his "Examination of Reviews, &c."*

Bristol, Nov. 16, 1841.

*My Dear Sir*,—Having just received from Dr. Paine a copy of his "Examination" of the Critique on his Medical and Physiological Commentaries, which appeared in the April number of the British and Foreign Medical Review, I find, to my great surprise, that Dr. P. has thought himself justified—not only in singling *me* out as the author of it, and in animadverting upon what he considers to be *its* misrepresentations, as if they were *mine*, (thereby attempting to make that a matter of personal discussion between us, for which the editor of the Review holds himself responsible)—but also in fixing upon me a charge of literary plagiarism, which is calculated, if I allow it to remain uncontradicted, to do great injury to my personal as well as to my scientific character.

Before going further, I must express my astonishment that any person holding the position which Dr. Paine occupies, should commit himself to so grave a charge against an individual, to whose discredit he *knows* nothing, upon evidence so flimsy as that which he adduces; especially as he must have been aware that, from the distance of the accused party, his defence could not be laid before the public until many months should have elapsed since its publication, during which time an injurious impression would have been formed not easily to be eradicated. And I think that I have further a just right to complain, that Dr. Paine's inculpation of me is not confined to surmise; but that, after he has proved his point to his own satisfaction, he has taken it for granted and, throughout the latter part of his pamphlet has continually coupled my name with the accusation of gross plagiary.

The evidence which Dr. P. adduces in support of the charge, is briefly the following:—Having made up his mind, from certain coincidences of opinion and of expression, between the Critique on his Commentaries, and my Principles of Physiology, that I must be the writer of the former, he has searched in previous numbers of the same Review for articles written, as he imagines, by the same author. In this search he thinks himself assisted by references occasionally made from one article to another,—the complete fallacy of which kind of evidence is exposed in Dr. Forbes's letter. Upon the same evidence, I must have been the reviewer of my own work; and I am not certain whether Dr. P. does not mean to insinuate as much. Any person, however, who carefully reads that review, which I did not see until it was in print, may find abundant evidence of the absurdity of such an idea. With respect to the other chief source of Dr. P.'s evidence,—coincidence in opinion, and in the mode of expressing it,—I will only say that Dr. P. shows great ignorance of the state of physiological science in this country, if he imagines that the opinions expressed in my Principles, on the subjects alluded to, are at all peculiar to myself, and it is very natural that one writer should almost unconsciously adopt the phraseology of another who has recently treated of the same questions, when desiring to express the same ideas.

So much for the evidence on which Dr. P.'s charge is founded. I have thus examined it, merely to show how unjustifiable it was in Dr. P. to charge me with the perpetration of a gross literary theft, upon no better grounds. The charge itself,—that, in a review of Hunter on the Blood, in a former volume of the same Journal, I unceremoniously adapted certain passages from Dr. Channing's Essay on Milton, to a very different purpose,—is easily disposed of. *I did not write that review.* To those who know me, my simple denial would, I am confident, be amply sufficient; but for the satisfaction of Dr. Paine, who, in his ignorance of my character, may think me as capable of asserting a falsehood as of stealing a paragraph, I enclose a note from Dr. Forbes confirmatory of my assertion.

Dr. Paine considers that his identification of me with the plagiarist is triumphantly confirmed, by a correspondence which he imagines that he has detected, between certain passages in my Principles of Physiology, and others which he has selected from Dr. Channing's Sermons. I am myself completely at a loss to discover this correspondence; and my friends here find it equally difficult. The falsity of this charge is as easily proved as that of the other; for *I have never* (I speak it almost with shame) *read the Sermons* from which Dr. P. quotes. The ideas which I have expressed have so long been familiar to my mind, that I cannot imagine that they involve any thing peculiarly *Channing-ian.* If any correspondence do exist, it is easily accounted for by the fact, that I received my education from one who was for many years the respected and attached friend of that illustrious man, and whose mind, cast in the same mould with his, impressed mine with those habits of thought, which have led to whatever similarity may present itself between our published opinions.

In regard to Dr. Paine's criticisms upon the scientific opinions I have expressed in my Principles of Physiology, I shall not now offer any remarks; nor do I intend to take up the guantlet from an opponent, who has shown himself so destitute of judgment and of good feeling. Of the merits of our respective productions I am quite content to leave the public to judge.

Having few means of placing my statement before the medical public of America, save through your mediation, I take the liberty of so far trespassing on your kindness, as to request you to gain insertion for it in such journals, as may give it a circulation equal to that of Dr. Paine's calumnious charges against me.

Believe me to remain, dear Sir,
Respectfully and sincerely yours,
WILLIAM B. CARPENTER.

*From Dr.* FORBES, *Editor of the British and Foreign Medical Review, to Dr.* W. B. CARPENTER.

DEAR CARPENTER,—As I think it would be a piece of silliness, only second to that of writing and publishing the "Examination," to attempt any detailed or serious reply to Dr. Paine's wordy reclamation, or any justification of the article in the Review to which it refers, I shall take no notice whatever of his attack, further than relates to the charge of plagiarism. *This is true*, so far as the writer of the review on Hunter is concerned, but *false* as concerns *you*,—since you did not write that review. This I am ready to state to all persons, at all times, as the truth, without any reservation or equivocation. The conduct of the writer of that review, in palming upon the Editor a portion of the writings of another for his own,—if really done intentionally and with a view to deceive (I would fain hope that the fact may admit of some other interpretation,) cannot be sufficiently reprobated. Although, as being the first specimen I had had of this person's writing (and, with one trifling exception, the only one I have ever had) I might be forgiven for not suspecting the authenticity of the surreptitious passages, I take shame to myself for being so little acquainted with the eloquent writings of Dr. Channing, as not to detect the theft before the MS. left my hands for the press.

Perhaps when Dr. Paine discovers that he is mistaken in the affiliation of this portion of the Review, he may feel somewhat less confident of the evidence by which he thinks he has traced the authorship of other articles in it to you. I certainly shall not gratify his curiosity on this point, by either affirming or denying the accuracy of his conclusions; and I do not see any reason why you should.

It is singular that Dr. Paine should have been so ignorant of the ordinary mode of conducting a Review, as not to know that the reference from one article to another is no proof whatever of the identity of the authorship of the two,—even when this reference is made by the writer of the latter article. But, most commonly, such references are made by the editor, without any communication with the original writer, in the exercise of the privileges inherent in the office of the great editorial WE.

In looking at the vast accumulation of words in Dr. Paine's pamphlet, I confess that I feel regret that the review of his book (just and accurate as I still hold it to be) was not more favourable; as it is melancholy to think that so much time and pains should have been stolen from tasks of usefulness, and expended in elaborating a work which, of course, no human being will read, except the author himself, perhaps the writer of the inculpated article, and, alas! the editor of the review.

It is lamentable to see how this mortification of Dr. Paine's self-love has clouded his judgment throughout the whole composition of his pamphlet; and this obfuscation is nowhere more conspicuous than where he attempts to convict you of plagiarising, in your "Principles of Physiology," from Dr. Channing. The very examples he adduces confute the charge.

Believe me, dear Carpenter, to be
Most truly yours,
JOHN FORBES.

*Old Burlington Street*, Nov. 15, 1841.

